# The association study of genetic variants with developing musical aptitude in humans

**DOI:** 10.18699/vjgb-26-51

**Published:** 2026-05

**Authors:** A.V. Kazantseva, A.V. Toropova, E.K. Khusnutdinova, S.B. Malykh

**Affiliations:** Institute of Biochemistry and Genetics – Subdivision of the Ufa Federal Research Centre of the Russian Academy of Sciences, Ufa, Russia Ufa State Petroleum Technological University, Russia; Moscow Pedagogical State University, Moscow, Russia; Institute of Biochemistry and Genetics – Subdivision of the Ufa Federal Research Centre of the Russian Academy of Sciences, Ufa, Russia; Psychological Institute of the Russian Academy of Education, Moscow, Russia M.V. Lomonosov Moscow State University, Department of Psychology, Moscow, Russia

**Keywords:** music, logistic regression, mathematical model, cognitive abilities, α-synuclein, dopamine, thickness of Heschl’s gyrus, музыка, логистическая регрессия, математическая модель, когнитивные способности, α-синуклеин, дофамин, толщина извилины Хешля

## Abstract

The development of musical abilities, including absolute pitch, musical memory, rhythm sense, and musicality, at a high degree is determined by a hereditary component (up to 68 %). The studies implementing a genome-wide linkage and association approach to musical aptitude have revealed more than 100 genetic loci. This spectrum is comprised of the genes encoding for transcription factors and those responsible for neurogenesis and synaptic plasticity, genes fixed as a result of positive selection of musicality, and those related to inner ear formation. Since no studies linking musical aptitude with genes have been previously conducted in Russia, the present study aimed at replicating the association of 17 previously identified genetic variants with developing musical abilities in Russians. Genotyping of SNPs in the GATA2, PCDH7, UNC5C, ASAP1, SBSPON, DCBLD2, KALRN, VLDLR, OTOF, GRIN2B, FoxP1, FoxP2, BDNF, EGR1, and SNCA genes was performed using competitive allele-specific PCR in a sample of students who underwent rigorous contest selection at admission to the conservatory and in the corresponding control group. A series of logistic regression analyses were used both to evaluate the main effect of SNP and to identify the best prognostic model based on various loci. The mathematical model obtained by including only statistically significant SNPs consisted of GATA2 rs9854612, SNCA rs356168, rs3910105, ASAP1 rs3057, and VLDLR rs1454626 (р = 0.0018, pseudo r2 = 0.188, AUC = 0.791). The addition of all examined SNPs as predictors enabled the construction of a statistically significant model with a higher predictive ability (р = 0.012, pseudo r2 = 0.380, AUC = 0.889). The results revealed indicate a potential cumulative gene effect, confirming the involvement of dopaminergic and GABAergic neurotransmission, the reelin pathway and the role of alpha-synuclein in musicality formation.

## Abstract (ru)

Формирование музыкальных способностей, включающих абсолютный слух, музыкальную память, чувство ритма, музыкальность, в значительной степени определяется наследственной составляющей (до 68 %). Проведенные к настоящему времени работы с использованием полногеномного анализа сцепления и ассоциаций с музыкальной одаренностью позволили выявить более 100 генетических локусов. В этот спектр входят гены транскрипционных факторов, регуляции нейрогенеза и синаптической пластичности; гены, закрепленные в ходе позитивной селекции музыкальности, а также связанные с особенностями формирования внутреннего уха. Поскольку ранее в Российской Федерации исследований по изучению связи музыкального таланта с генетической компонентой не проводилось, настоящая работа направлена на репликацию ассоциации ранее идентифицированных 17 однонуклеотидных вариантов (SNP) с формированием музыкальных способностей у русских. Генотипирование полиморфных локусов в генах GATA2, PCDH7, UNC5C, ASAP1, SBSPON, DCBLD2, KALRN, VLDLR, OTOF, GRIN2B, FoxP1, FoxP2, BDNF, EGR1, SNCA проводилось с помощью конкурентной аллель-специфичной ПЦР в выборке студентов, прошедших строгий конкурсный отбор при поступлении в консерватории, и в соответствующей контрольной группе. Метод логистической регрессии применялся как для оценки основного эффекта отдельных полиморфных вариантов, так и для выявления наилучшей прогностической модели, содержащей различные генетические локусы. Математическая модель, полученная в результате включения только ассоциированных SNP, состояла из генетических локусов GATA2 rs9854612, SNCA rs356168 и rs3910105, ASAP1 rs3057 и VLDLR rs1454626 (р = 0.0018, псевдо-r2 = 0.188, AUC = 0.791). Добавление всех изученных генетических локусов в качестве предикторов в регрессионный анализ позволило создать статистически значимую модель, обладающую более высокой прогностической способностью (р = 0.012, псевдо-r2 = 0.380, AUC = 0.889). Полученные результаты указывают на потенциальный кумулятивный эффект белковых продуктов изученных генов, подтверждая вовлеченность дофаминергической и ГАМКергической нейротрансмиссии, рилинового пути и роль альфа-синуклеина в формировании музыкальности.

## Introduction

Music is an integral part of cognitive and social interaction between humans, which was used as an important component in the information transfer even before the speech formation. It is assumed that music existed more than 35,000 years ago, evidence of which is found in archaeological excavations of caves in Germany, Austria, and France (Conard et al., 2009). Despite the fact that currently this function mainly belongs to verbal communication, musical abilities are closely related to cognitive and mental activity. Musical abilities cover a significant range of various individual psychological characteristics, including absolute hearing, musical memory, rhythm sense, and musicality (Aikina, 2017), and represent quantitative parameters, the distribution of which in a population corresponds to the law of normal distribution.

The ability to perceive and reproduce music is assumed to be caused by a hereditary component, which initiated research seeking to identify the genes responsible for the development of musical abilities (musical talent). The results from twin studies as the primary stage of examining the role of genes and the environment in the formation of behavioral and cognitive abilities (Kazantseva, 2008) indicate a significant contribution of the hereditary component: genes determine up to 68 % of variance in musical aptitude (Oikkonen et al., 2015).

Some of the pioneer studies in the field of genetics of highlevel musical aptitude were based on the examination of largesized pedigrees of Finnish origin, which were characterized by accumulated skills of musical aptitude in families (Pulli et al., 2008). Using the genome-wide linkage approach (GWLS), the abovementioned and other research teams succeeded in identifying chromosomal regions 3q21.3 (including the GATA2 gene), 4p15.1 (including the PCDH7 gene), 4q22 (including the UNC5C gene), 8q24.21 (including the ASAP1 gene), and 8q21.11 (including the SBSPON gene) as linked with specificity of music perception (Pulli et al., 2008; Oikkonen et al., 2015) and absolute pitch (Theusch et al., 2009).

Published genome-wide association studies (GWAS) identified genetic loci, which determine individual variance in thickness of Heschl’s gyrus, i. e., the central region of the auditory cortex, responsible for speech and music perception, including the DCBLD2 rs72932726 and the KALRN rs333332 (Cai et al., 2014). On the other hand, GWAS enabled the detection of SNPs related to positive evolutionary selection of musical abilities (Liu et al., 2016). Namely, the study of Finnish respondents with high-degree musicality resulted in the detection of several genomic regions, including the VLDLR, FOXP1, OTOF, and GRIN2B genes, which formed the haplotypes being statistically more frequent among musically talented persons. The involvement of the abovementioned genes is unsurprising, since specific changes in their nucleotide sequences have been previously linked with speech impairment in humans (Rappold et al., 2023), vocalization and changes in gene expression in songbirds (Adam et al., 2016; So et al., 2019; Heim et al., 2023).

A development of any quantitative trait is known to be caused by the effects of multiple genetic loci, which requires research devoted to musical abilities to be conducted using data on a large number of genes and their structural variants. Therefore, together with GWAS and GWLS data, the results of functional studies, which were carried out with the use of model animals and enabled the estimation of the expression levels of annotated genes, are of great interest. In particular, a differential expression of the FoxP2, BDNF, and EGR1 genes was observed in songbirds during learning and vocalization and in model mice via listening to music (Li et al., 2010; Drnevich et al., 2012; Shi et al., 2013). Moreover, changes in the expression patterns of the alpha-synuclein gene (SNCA), the mutations of which are linked to predisposition to Parkinson’s disease and other synucleinopathies (Järvelä et al., 2018), were determined in humans as a result of listening to classical music (Kanduri et al., 2015).

Within the present study we have selected SNPs in the genes related to the development of musical abilities and characterized by changes in gene expression observed via listening to music. Gene polymorphisms were obtained either directly from the GWAS and GWLS or – in case information on certain SNPs being linked with musical abilities was absent – from the studies reporting the impact of genetic loci on the development of related behavioral traits (Crawford et al., 2008; Hu et al., 2011; Koks et al., 2021). The final information on the genes and their genetic variants selected as the objects of the present study is provided in Table 1.

**Table 1.**
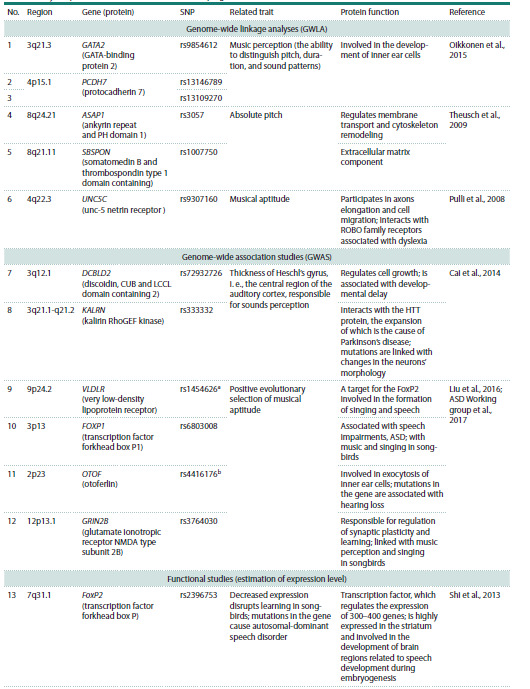
Polymorphic variants associated with developing musical abilities

**Table 1end.**
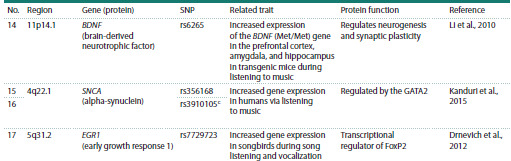
Table 1end. Note. We have selected polymorphisms in genes with a previously reported link with: a lipoprotein levels (Crawford et al., 2008); b social abilities in patients with autism spectrum disorder (ASD) (Hu et al., 2011); c differential gene expression caused by various allelic variants (Koks et al., 2021).

The present study represents the extension of our previous research, which estimated a probability to develop high-level musical aptitude based on 10 genetic variants (Kazantseva et al., 2023). Accordingly, the present research is aimed at replicating the involvement of the extended panel of genetic variants in developing musical talent in ethnic Russians from the Russian Federation.

## Materials and methods

The experimental part of the study included a selection of individuals characterized by musical talent who were students at conservatories (Moscow) and passed a severe competitive contest upon admission, during which they demonstrated outstanding abilities in entrance exams on solfeggio, harmony, polyphony, reading scores, etc., depending on the chosen specialty.

The sample consisted of ethnical Russians aged 18–22 years (N = 100, 66 % women; mean age 19.36 ± 1.44 years). The control group was comprised of Russian students at the Universities of Moscow and Ufa corresponding by age and sex to the sample of musicians (N = 200, 67 % women; mean age 19.84 ± 1.85 years). All the examined subjects were not observed in neuropsychiatric institutions and denied a family history of mental disorders. Voluntary consent to participate in the study was obtained from all the respondents. The study was approved by the Bioethical Committee at the Institute of Biochemistry and Genetics of UFRC of RAS.

A collection of biological material (5 ml saliva) was carried out in the tubes containing a preserving agent according to the manufacturer’s recommendations (Oragene DNA, DNA Genotek, Canada). Subsequent DNA isolation was performed using specific kits (PrepIT, DNA Genotek, Canada), followed by the assessment of DNA quality via spectrophotometer. Genotyping of examined SNPs was conducted via endpoint real-time PCR on the CFX96 DNA Analyzer (BioRad, USA) using KASP chemistry (LGC Genomics, UK).

A correspondence of observed genotype frequencies distribution to the theoretically expected one based on the Hardy–Weinberg law was carried out in the control group and revealed a deviation of the PCDH7 rs13109270; therefore, this SNP was excluded from subsequent steps of the analysis. Statistical analysis was based on testing of various regression models of analyzed genetic loci, including additive, dominant, recessive, overdominant, and codominant (PLINK v.1.09).

The use of the additive model demonstrates whether the effect of the alternative allele is accumulative; the dominant model shows whether the effect of the tested allele is observed in the presence of at least one copy (i. e. in the heterozygous state and in the homozygote of the effect allele); the recessive model shows whether the effect of the examined allele is prominent with the presence of both copies. The overdominant model describes a state when the highest effect is observed when both alleles are present (i. e. in the case of a heterozygous genotype) compared with both reference or alternative alleles (i. e. in the case of any homozygous genotype). The codominant model assumes that any genotype demonstrates a significant effect on developing the examined trait in a non-additive manner, i. e., independently of other genotypes (Kutikhin et al., 2017).

Multiple logistic regression analysis was used to test for different models (including all the SNPs) and to design the final mathematical model, which included statistically significant predictors and explained the highest proportion of variance in developing high musical aptitude (R v.4.4.2). To estimate the models’ quality, the Akaike information criterion (AIC) was used: the lower its value, the higher the model’s quality. The best model was visualized as the ROC (Receiver Operating Characteristic) curve reporting a quantitative measure of the AUC (area under curve) parameter.

## Results

At the first stage of association analysis between genetic variants and high-level musical aptitude, we confirmed the involvement of the ASAP1 rs3057 (р = 0.032) in developing musical abilities at a statistically significant level (Table 2). In particular, enhanced chances of demonstrating musical abilities were characteristic of heterozygous carriers of the ASAP1 rs3057 С/Т genotype compared with homozygotes (T/T and C/C genotypes) (OR = 1.94, 95 % CI 1.05–3.56), which stands in evidence of a higher effect of the heterozygous genotype of this SNP exactly on manifesting musical talent. Other genetic variants failed to demonstrate an association with musicality at a single-locus level in any analyzed model: the additive, dominant, recessive, and deviation from dominance models.

**Table 2.**
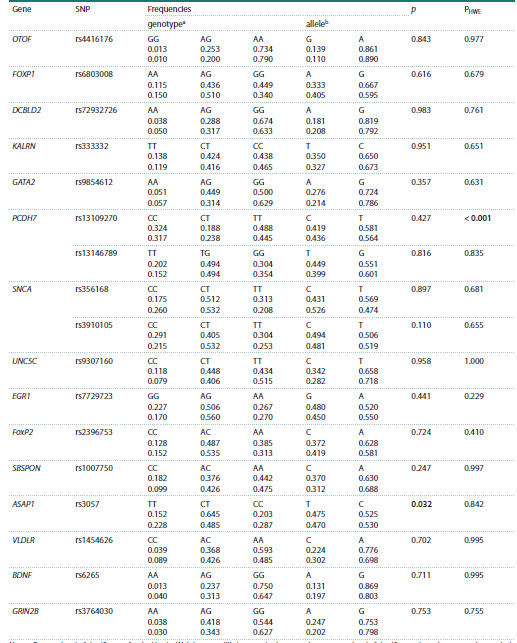
Allele and genotype frequencies of examined polymorphisms in the group of musicians and in the control group and the results of association analysis Note. PHWE – a level of significance for the Hardy–Weinberg equilibrium test in the control group; р – a level of significance based on regression analysis is given for comparative models between a heterozygous genotype with a combined sample of homozygotes (overdominant model); genotype (a) and allele (b) frequencies are reported for the group of musicians (upper lane) and the control group (bottom lane), respectively. Statistically significant differences are marked in bold.

Since the development of high-level musical aptitude, like any other complex trait, represents a result of the interaction of proteins encoded by various genes, the subsequent stage was based on a series of multiple regression analyses, which consisted of 16 SNPs as predictors at the initial level (model 1 (Table 3) χ2 = 46.24, р = 0.012). After the exclusion of statistically insignificant SNPs, we obtained a model that included the GATA2 rs9854612, SNCA rs356168 and rs3910105, ASAP1 rs3057, and VLDLR rs1454626 gene polymorphisms (model 2 (Table 3) χ2 = 24.61, р = 0.0018).

**Table 3.**
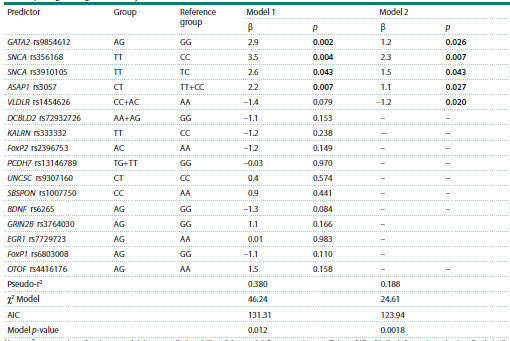
Predictive models of liability to developing high-level musical abilities detected as a result of multiple logistic regression analyses Note. r2 – proportion of variance explaining a predictive ability of the model; β – regression coefficient; AIC – Akaike information criterion. Statistically significant differences are marked in bold.

According to the final model, the highest chances of manifesting musical talent will be realized in the case of simultaneous presence of the following genotypes: the GATA2 rs9854612 A/G (р = 0.026), SNCA rs356168 T/T (р = 0.007) and rs3910105 Т/Т (р = 0.043), ASAP1 rs3057 С/Т (р = 0.027), and VLDLR rs1454626 А/А (р = 0.027).

Therefore, the highest genetic effect linked to developing musical abilities will be observed in the case of the presence of a heterozygous variant compared with the carriers of any homozygote (ASAP1 rs3057, overdominant model) or the carriers of both copies of the alternative allele (GATA2 rs9854612, codominant model).

The results of logistic regression also point to the potential positive accumulated effect of the T allele (in both SNPs of the SNCA gene: rs356168 and rs3910105, recessive model, the effect will be observed only in the presence of both alleles of this gene) and the C allele (VLDLR rs1454626, dominant model, it will be evident in the presence of at least one copy of the effect allele) on musical talent.

Construction of ROC curves and the calculated area under curve (AUC) testify that model 1, which consists of all 16 SNPs, has the highest prognostic ability (AUC = 0.889) (see the Figure), although not all included predictors demonstrate a statistically significant effect on developing musical abilities. Despite the lower predictive ability of the final model (model 2) (AUC = 0.791) (see the Figure), it is characterized by better quality parameters (AIC = 131.31 for model 1, AIC = 123.94 for model 2).

**Fig. 1.**
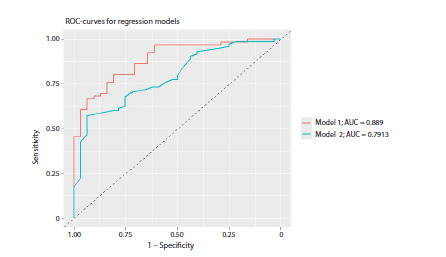
The results of modeling ROC curves for logistic regression models. Including various combinations of the examined genetic loci as predictors (model 1 contains all 16 SNPs; model 2 contains five most significant SNPs in the GATA2, SNCA, ASAP, and VLDLR genes). A detailed description of variables included in the models is given in Table 3. The values of areas under the curves (AUC) for each model are reported in the legend.

## Discussion

Within the framework of the present study, we carried out a replication association analysis of gene polymorphisms, which had previously demonstrated their relation to musical aptitude and music perception. The inclusion of all examined genetic variants as predictors in the regression analysis enabled the construction of the statistically significant model with high predictability (area under the ROC curve = 0.889). However, the final model consisting of the data from five gene polymorphisms, i. e. the GATA2 rs9854612, SNCA rs356168 and rs3910105, ASAP1 rs3057, and VLDLR rs1454626, demonstrated more statistical significance and better quality (area under the ROC curve = 0.791).

**Genetic loci identified in GWLA**

In the present study we have analyzed genetic loci rs9854612, rs13146789, and rs13109270 located in proximity to the GATA2 and PCDH7 genes, which demonstrated a linkage with the specificity of music perception at a genome-wide level (Oikkonen et al., 2015). It is known that GATA binding protein 2, encoded by the same gene, is involved in the development of inner ear hair cells, which are important for tonotopic mapping (Oikkonen et al., 2015). Moreover, GATA2 participates in dopaminergic neurotransmission and regulates the expression of the alpha-synuclein gene (SNCA), the enhanced transcription of which is observed together with dopamine release during listening to music (Järvelä, 2018), pointing to the involvement of the brain reward system in this process.

The present study demonstrated a positive effect of the simultaneous presence of single copies of reference and alternative alleles in the GATA2 rs9854612 gene polymorphism on increased chances of developing musical talent. In turn, protocadherins regulate neuronal migration, differentiation, and synaptogenesis and participate in the formation of cochlear-nuclear and amygdaloid complexes (Oikkonen et al., 2015), promoting their impact on musical abilities. The opposite effect was mentioned during a segregation analysis of a deletion of a cluster of protocadherin genes in the 5q31.1 genomic region in large pedigrees, which resulted in reduced musical creativity (Ukkola-Vuoti et al., 2013).

Interesting data were obtained in a recent study indica- ting a link between plasma hypermethylation of the gene cluster, including PCDH7, and lead concentration in the blood of pregnant women whose previous pregnancy resulted in the birth of an ASD child (Aung et al., 2022). Based on gene ontology analysis provided by the authors, the level of this toxic metal is related to such biological processes as neuronal development and changes in the immune system regulation, which may cause abnormal development of the fetal nervous system. Since no findings on a functional role of allelic variants of the examined SNPs in the GATA2 and PCDH7 genes were published to date, it cannot be unambiguously concluded that a differential expression of these genes accompanies the formation of musical talent in the examined sample of Russians

The assessment of a single-locus effect resulted in the detection of a statistically significant increase in the ASAP1 rs3057 С/Т genotype frequency in students with high-degree musical abilities compared with the control group, which assumes the existence of an overdominant model of inheritance characteristic for this SNP, since the highest positive effect was observed in the case of the simultaneous presence of both reference and alternative alleles. The examined gene polymorphism is known for its regulatory effect (Bryzgalov et al., 2013) and was previously detected as linked with absolute pitch (LOD = 3.46 in Europeans) (Theusch et al., 2009).

Existing data signify that the ASAP1 gene is one of 30 genes differentially expressed in the prefrontal cortex as a response to ADHD treatment (dela Peña et al., 2014), indicating its relevance to the regulation of cognitive processes. In addition, published findings reported a link between other polymorphisms in the ASAP1 gene with behavioral characteristics in normal and pathological states, including schizophrenia (Goes et al., 2015), cognitive decline (Mega Vascular Cognitive Impairment and Dementia (MEGAVCID) consortium, 2024), and education level (Okbay et al., 2022).

The examined SBSPON rs1007750 was initially identified as linked with absolute pitch segregation (Theusch et al., 2009) and, in addition, was present in a set of differentially expressed genes in postmortem tissues of the prefrontal cortex of schizophrenia patients (Maycox et al., 2009).

Another genetic variant, which was included in a prognostic model of musical talent, is rs9307160 located in the netrin receptor gene (UNC5C), which was linked with accumulation of musicality in Finnish families (Pulli et al., 2008). The netrin receptor UNC5C is expressed in dopaminergic neurons and participates in netrin signal transfer within neuronal migration during development and axonal guidance (Treccarichi et al., 2024). Previous research evidences a relation of certain mutations in the UNC5C gene to developing familial forms of Alzheimer’s disease, schizophrenia, bipolar and depressive disorders, and other mental disorders (Treccarichi et al., 2024) due to shifted neuronal sensitivity to apoptosis.

Preserved cognitive functioning at the elderly age is related to polymorphisms in the UNC5C gene as well as to changes in its methylation and diminished levels of its expression in the dorsolateral prefrontal cortex, which predominantly mediates the changes in the presynaptic terminal (White et al., 2017). Despite the unknown functional significance of the rs9307160 in the regulation of the UNC5C gene expression, the abovementioned findings indicate its potential involvement in the development of both cognitive and behavioral reactions mediated by dopaminergic neurotransmission.

**Genetic loci identified in GWAS**

Genetic variants in the VLDLR, FOXP1, OTOF, and GRIN2B genes, which were included in our regression model, have been initially identified as connected with positive evolutionary selection of musical abilities (Liu et al., 2016). The VLDLR gene encodes low-density lipoprotein receptor involved in the reelin signaling cascade and representing the FoxP2 target. The latter is a transcription factor playing a significant regulatory role in vocalization and speech formation. In particular, a knockout of the FoxP2 gene promotes a decline in the level of target VLDLR protein in model animals, whereas an enhanced expression of the VLDLR gene was determined in specific brain region of songbirds during learning and vocalization (Adam et al., 2016), which points to a potential role of the reelin signaling cascade in cognitive processes related to musicality. In addition, increased expression of the genes controlled by FoxP2 was observed in human peripheral blood as a result of listening to music (Kanduri et al., 2015).

According to previous research, the selected VLDLR rs1454626 gene polymorphism (C allele) is associated with enhanced lipoprotein level and body mass index; interestingly, this effect was strengthened with a simultaneous presence of the “risky” ε4 variant in the apolipoprotein E (APOE) gene (Crawford et al., 2008). The data obtained by our groups on the association of the rs1454626 C allele with a lower probability to develop musical abilities coincide with representations of the link between cognitive functioning and high-density lipoprotein-to-low-density lipoprotein ratio (Poliakova, Wellington, 2023) and tend towards a dominant model of inheritance of a negative effect of the C allele on developing high-level musical aptitude.

Transcription factor genes examined within the present study, i. e. FoxP1 (rs6803008) and FoxP2 (rs2396753), regulate the expression of a high number of genes, including reelin pathway genes (VLDLR). De novo mutations in the FoxP1 gene underlie the etiology of FoxP1 syndrome – an autosomal-dominant disease characterized by abnormalities in the cognitive and language abilities, communication, and behavior, including ASD, ADHD, anxiety disorders, and oromotor dysfunctions (Rappold et al., 2023). Moreover, FoxP1 mediates vocal preferences and motivation for active listening to bird singing in female zebra finches (Heim et al., 2023), which may indicate a potential role of this transcription factor in the formation of human musicality.

Another significant member of the processes of cognitive functioning and synaptic plasticity is the glutamatergic system, an important component of which is the GRIN2B gene encoding the glutamate receptor subunit, examined in the present study. An increase in the mRNA level of this gene was recorded as a result of songbird vocalization (in particular, zebra finches) (So et al., 2019). According to published studies, the examined allelic variant rs3764030 (А allele) in the GRIN2B gene was also associated with improved information processing speed due to enhanced gene expression (Jiang et al., 2017). Based on our findings, the rs3764030 A allele is linked with a positive prognostic effect toward musical abilities under the framework of the regression model, which is consistent with the abovementioned data.

The otoferlin gene was also identified as one with a selective advantage for evolutionary consolidation of musical abilities (Liu et al., 2016). This gene encodes a transmembrane protein involved in the exocytosis of the inner ear cells, indicating its potential involvement in musicality. Since no previous research demonstrated an association of a distinct polymorphism in the OTOF gene with musical traits, we have selected rs4416176, since its allelic variants had an earlier reported association with social abilities in ASD patients (Hu et al., 2011).

The role of the OTOF in hearing impairment gene has been repeatedly examined, whereas the introduction of the OTOF gene therapy in children with congenital deafness demonstrated a pronounced positive effect on hearing restoration, improvement of auditory and speech abilities, and music perception, even compared with patients with cochlear implantation (Cheng et al., 2025).

Genetic loci in the DCBLD2 (rs72932726) and KALRN (rs333332) genes have been selected based on GWAS results as associated with the thickness of Heschl’s gyrus (Cai et al., 2014) – an auditory cortex region involved in sounds and speech perception, linguistic tone, and regulating inner ear development. It should be noted that musicians are characterized by exaggerated gray matter volume in the right and left parts of the auditory cortex, while an increase in the right Heschl’s gyrus is typical for musicians with absolute pitch (Wengenroth et al., 2014). rs72932726 in the DCBLD2 gene, which was introduced in the final regression model in the present study, is also linked with the structural changes in brain regions related to speech and language processing (Cai et al., 2014).

Another examined gene is KALRN, encoding kalirin kinase – a synaptic regulator, which is responsible for dendrite morphogenesis, axonal growth, and brain connectivity (Parnell et al., 2021). Impaired regulation of the KALRN gene was observed in cognitive deficit, schizophrenia, and ASD (Parnell et al., 2021), while the genetic variant rs333332 in the KALRN gene demonstrated the association with the thickness of the left Heschl’s gyrus (Cai et al., 2014).

**Polymorphisms in differentially expressed genes**

The final regression model of liability to musical aptitude consisted of gene variants previously demonstrating functional changes in the level of gene expression. Namely, lower expression of the transcription factor FoxP2 gene, known for its important role in neuronal differentiation, brain and speech development, was observed in the case of impaired songbird vocalization (Shi et al., 2013), while structural changes in this gene were related to clinical symptoms of schizophrenia (Salmón-Gómez et al., 2025) and speech perception (Ocklenburg et al., 2013). In addition, based on a recent systematic review, the rs2396753 C allele was associated with a reduced gray matter density in the brain and was more frequent in individuals with verbal hallucinations accompanying schizophrenia (Salmón-Gómez et al., 2025). At the same time, our findings indicate the association of the rs2396753 C allele with absent musicality.

It is known that early growth response protein (EGR1) involved in neuronal plasticity and mediated by differential expression microRNAs miR-23a and miR-23b is a transcriptional regulator of the FoxP2 gene (Nair et al., 2021). Multiple studies point to the involvement of EGR1 protein in memory and learning, while its expression in different brain regions demonstrates a stress-inducible pattern, thus accompanying such mental disorders as schizophrenia, depression, and Alzheimer’s disease (Gallo et al., 2018).

Brain-derived neurotrophic factor (BDNF) is one of the most frequently analyzed genes in the context of cognitive abilities and disabilities involved in the regulation of neurogenesis and synaptic plasticity, enhanced expression of which is observed after listening to music (Li et al., 2010). According to model 1, the rs6265 (Val66Met) A/G (Met/Val) genotype is characterized by a lower odds ratio of developing musical talent compared with the G/G (Val/Val) genotype. Multiple studies evidence that the rs6265 Met variant (coded by the A alelle) is linked with reduced gene expression and diminished gray matter volume in brain regions participating in memory formation, self-control and emotional regulation (Kunikullaya et al., 2025).

Previously, a positive effect of musical listening on increased BDNF level was also determined, which promoted a decline in depressive symptoms (Yeh et al., 2015). Such dependence is suggested to be caused by the involvement of rs6265 in the regulation of neuroplasticity in the auditory cortex of the brain. The analysis of mismatch negativity (MMN) as a value reflecting the process of automatic error detection by the auditory cortex and preconception processes, which was conducted during electroencephalography, revealed that the most significant changes in MMN peaks were characteristic of musicians carrying the Val/Val genotype compared with musicians with the Met allele (Bonetti et al., 2023).

In turn, music perception affects changes in alpha-synuclein (SNCA) gene expression at the molecular level (Kanduri et al., 2015). Encoded protein is involved in regulation of dopamine biosynthesis via altering its reuptake from the synaptic cleft by transporter protein and demonstrates its link with the ethiopathogenesis of Parkinson’s disease (PD). At the SNPs level, existing data evidence the association of the SNCA rs356168 C/C genotype with increased risk of PD and an enhanced alphasynuclein level in CD45+ blood cells of patients (Emelyanov et al., 2018). It is assumed that rs356168 regulates a ratio between long (based on the 3´ region) and short transcripts of alpha-synuclein, therefore determining its ability to oligomerize and PD development (Rhinn et al., 2012).

In the present study we demonstrated the effect of alternative rs356168 and rs3910105 T/T genotypes on musical talent within a recessive model of inheritance, which suggests that a favorable effect can be observed only in the presence of both copies of the T allele. The data obtained are unsurprising, since they coincide with conclusions published in a recent systematic review (Navarro et al., 2023) pointing to a link between dopaminergic neurotransmission, alpha-synuclein level, the positive effect of music listening and reproduction (Kanduri et al., 2015; Järvelä, 2018), and clinical manifestations of neurodegenerative diseases.

Moreover, the obtained evidence on a significant cumulative effect of the examined genes coincides with the data of a meta-analysis (Kunikullaya et al., 2025), which confirmed the role of brain-derived neurotrophic factor, alpha-synuclein, and GATA2 in modified neurogenesis and neuromediation caused by music listening and reproduction. The last one initiates exaggerated levels of neurotrophins, neuroprotectors, and synaptic plasticity regulators, improves immune functions, and reduces stress.

The conducted study has several limitations. Firstly, the analyzed sample has a small size; nevertheless, it is homogeneous by age, ethnic origin, and level of musical education. Secondly, the number of the studied genetic variants is limited and should be expanded for future research. In addition, several genetic loci were taken based on literature data on their association with similar phenotypes without information on their functional significance. Thirdly, despite the multifactorial nature of musical abilities, we had no data to conduct mathematical modeling accounting for potential environmental predictors such as the qualitative and quantitative specifics of musical education in childhood, the presence of a musical environment, socioeconomic status, etc. Fourthly, it is necessary to consider the pilot character of the present study and insufficient statistical power in the analysis of the predictive mode, including 16 SNPs, which may reflect the presence of type I and II errors.

Further research in this field should focus on expanding the panel of examined genetic loci to study the predisposition to high-level musical abilities and assessing their functional significance in the context of regulating gene expression.

## Conclusion

Within the framework of replication association analysis, using a set of genetic loci previously identified in European samples, we constructed a prognostic model including five SNPs estimating a liability to musical aptitude in Russians at a significant level of sensitivity and specificity

Despite polymorphic loci being selected based on various approaches (GWAS, GWLS, differential expression data), the examined genes frequently overlap, and encoded proteins are frequently included in the same molecular pathways. Moreover, a significant part of the examined genes encode transcription factors, pointing to a potential impact of various molecular pathways regulated by these factors in developing musical abilities.

The data obtained indirectly evidence the interaction of the examined genes, confirming that musical aptitude is related to the specificity of dopaminergic and GABAergic neurotransmission, synaptic plasticity, neurogenesis, and reelin signaling cascade. Moreover, a commonality of molecular genetic factors involved in the ethiopathogenesis of schizophrenia, cognitive impairments, ASD, affective disorders, and musical talent was determined.

## Conflict of interest

The authors declare no conflict of interest.
